# Paraquat Exposure of Pregnant Women and Neonates in Agricultural Areas in Thailand

**DOI:** 10.3390/ijerph15061163

**Published:** 2018-06-03

**Authors:** Pajaree Konthonbut, Pornpimol Kongtip, Noppanun Nankongnab, Mathuros Tipayamongkholgul, Witaya Yoosook, Susan Woskie

**Affiliations:** 1 Department of Occupational Health and Safety, Faculty of Public Health, Mahidol University, 420/1 Rajvithi Road, Bangkok 10400, Thailand; pajaree.kon@mahidol.ac.th (P.K.); noppanun.nan@mahidol.ac.th (N.N.); 2 Department of Epidemiology, Faculty of Public Health, Mahidol University, 420/1 Rajvithi Road, Bangkok 10400, Thailand; mathuros.tip@mahidol.ac.th; 3 Faculty of Public Health, Mahasarakham University, Khamriang Sub-District, Kantarawichai, Maha Sarakham 44150, Thailand; wittaya.yoo@mahidol.ac.th; 4 Department of Public Health, Zuckerberg College of Health Sciences, University of Massachusetts Lowell, One University Ave, Lowell, MA 01854, USA; Susan_Woskie@uml.edu

**Keywords:** agriculture, paraquat, pregnant women, meconium, Thailand, herbicide, prenatal exposure

## Abstract

This study aimed to assess paraquat concentrations in the urine of women at 28 weeks of pregnancy, delivery and 2 months postpartum and in the meconium of neonates. In all, 79 pregnant women were recruited from three hospitals located in agricultural areas in Thailand. The subjects were interviewed about personal characteristics, agricultural activities and pesticide use patterns. Paraquat was analyzed in urine and meconium using high performance liquid chromatography equipped with a fluorescence detector. The geometric mean (GSD) of urinary paraquat concentrations at 28 weeks of pregnancy, delivery and 2 months postpartum were 2.04 (4.22), 2.06 (5.04) and 2.42 (5.33) ng/mL, respectively. The urinary paraquat concentrations at 28 weeks of pregnancy, delivery and 2 months postpartum between agriculturist and non-agriculturist were not significantly different (*p* = 0.632, *p* = 0.915, *p* = 0.57, respectively). The geometric mean (GSD) of paraquat concentration in the meconium was 33.31 (4.59) ng/g. The factors predicting paraquat exposures among pregnant women and neonates included working outside, living near farmland, having family members who work on a farm, drinking well water and using herbicides or paraquat.

## 1. Introduction

In Thailand, herbicides are heavily used in agriculture to protect crops and increase yields. The Thai Office of Agricultural Economics reported that in 2016, herbicides comprised the largest volume of imported pesticides (125,596 tons), followed by insecticides, fungicides and other crop protection products [1]. Among the herbicides, paraquat was the second most imported herbicide (31,525 tons) in 2016 [2]. Paraquat or gramoxone (1,1-dimethyl-4,4-bipyridnium) is a highly effective non-selective fast-acting contact herbicide [3]. Due to its high acute toxicity and adverse effects on human health, paraquat is now banned in over 50 countries, including the 27 countries of the European Union, Cambodia, China and Vietnam [3,4,5,6]. Paraquat is classified by WHO as a class II pesticide which is moderately hazardous to human health based on an oral LD_50_ in rats of 150 mg/kg body weight [7].

The toxicity of paraquat is related to its rapid reduction and subsequent re-oxidation producing reactive oxygen species. The accumulation of reactive oxygen species (ROS) and especially toxic free radicals in various organs, can result in paraquat poisoning [8]. Acute health effects occur frequently among paraquat users, including eye injury, nosebleeds, nail damage and skin irritation or burns. Chronic exposure to relatively low doses can affect the lungs, nervous system, brain, skin and eyes [3]. Additionally, a significant association between Parkinson’s disease and paraquat use was found in the U.S. Agricultural Health Study [9,10]. The Agricultural Health Study also showed evidence for a link between paraquat exposure among applicators and non-Hodgkin’s lymphoma and a slightly increased risk of breast cancer among women whose husband’s used paraquat [11,12].

Paraquat enters the human body by ingestion, inhalation and dermal contact and then is excreted unchanged in the feces and urine [3]. The main route of paraquat exposure for agricultural workers is skin contact because paraquat itself can make the skin more permeable, increasing dermal absorption [3]. Although dermal absorption is slow, fatalities have been reported when widespread absorption of paraquat through damaged skin has occurred [13]. Inhalation exposure of applicators was relatively low due to the low volatility and spray droplets too large to enter the small airway [13]. A study of paraquat exposures among sprayers on banana plantations in Costa Rica showed higher concentrations from dermal contact than inhalation [14].

Paraquat can cross the placenta and reach the fetus because of its relatively small diameter and low molecular weight [15]. In cases of attempted suicide during pregnancy, the paraquat level in fetal blood was found to be 4–6 times higher than that in maternal blood. Fetal deaths were reported to be due to paraquat reaching the organ tissues through blood circulation, leading to multiple organ failure [16,17]. Animal studies have shown that chronic prenatal exposure to paraquat, mancozeb fungicide or combinations of paraquat and mancozeb could cause impairment of motor coordination that may have longer term impacts on development [18]. The assessment of neonatal exposures to pesticides can be conducted using infant hair, cord blood and meconium [19]. Meconium has been used to measure fetal exposure to agents that pass across the placenta during gestation such as illicit drugs, heavy metals and pesticides [19,20,21]. Meconium is a very useful matrix to measure cumulative fetal exposures because it forms as early as the third month of gestation and accumulates until birth [19,20,22].

Paraquat is rapidly and strongly bound to soil; therefore, it is very stable in soil. The half-life of paraquat in soil is up to 20 years [3]. Paraquat could be bound to suspended or precipitated sediment in the aquatic environment. The half-life of paraquat in water is between 2 to 820 years depending on sunlight and depth of water [3]. Paraquat was found in surface water [23], drinking water [24] and in ground water [25]. The toxicokinetics of paraquat were studied in suicidal human cases, which indicated a mean distribution half-life of 5 h and elimination half-life of 84 h. Death related to pulmonary fibrosis was associated with the elimination phase [26]. The half-life in humans has not been studied or found.

Pregnant women living in agricultural areas may be exposed to paraquat through many pathways such as working in agricultural fields treated with paraquat or applying the herbicide, contaminants brought home from the agricultural fields by family members or consuming food with high residue levels. Previous studies of paraquat exposure have focused on occupational exposures during spraying activities [14,27,28,29,30,31,32], and case-reports from accidental exposures [27,33] or suicide [15,16,17,34,35,36] among the general population. To date, this is the first study to assess urinary paraquat concentrations among women living in agricultural areas during pregnancy, delivery and postpartum and also to assess paraquat concentrations in the meconium of neonates.

## 2. Materials and Methods

### 2.1. Study Population

Pregnant women were recruited from hospitals in three agricultural areas in Thailand: Amnatchareon Hospital in Amnatchareon Province, Sawanpracharak Hospital in Nakorn Sawan Province and Paholpolpayuhasena Hospital in Kanchanaburi Province. Data was collected from May 2011 to January 2012. At recruitment, women had to be in their 28th week of pregnancy, aged 19 to 35 years and without diabetes or hypertension. As this was part of a longitudinal study of child neurodevelopment, subjects were later excluded if they delivered infants at other hospitals, had pre-term labor, or birth by caesarian section.

### 2.2. Data Collection

At their 28th week of pregnancy and at 2 months postpartum, 79 pregnant women were interviewed about pesticide use at home during pregnancy, their home location, their sources of drinking water, history of work, agricultural activities during pregnancy, family members who are agriculturists living in the same house. If you are agriculturists you would answer about pesticides used in your own fields and you would be asking about the pesticides used by your family members living in the same house, if you know the answer. Urine samples were collected from the subjects at the 28th week of pregnancy, delivery and 2 months postpartum; the urine samples were collected randomly when the pregnant women came to the hospital according to their appointment. Meconium samples were collected from newborn infants by nurses within two day after delivery. Samples were kept frozen at −45 °C until analysis. This study was approved by the Ethics Committee on Human Rights Related to Human Experimentation, Mahidol University (MUPH 2011-098) and the University of Massachusetts Lowell Institutional Review Board (UML 10-129).

### 2.3. Analysis of Urine and Meconium Samples

Various method have been applied to analyze paraquat in urine, including gas-chromatography (GC), GC/mass-spectrometry (MS) [37,38] capillary electrophoresis [39], enzyme-linked immunosorbent assay (ELISA) [30], high performance liquid chromatography (HPLC) [40,41,42], HPLC/MS [43,44,45] and HPLC/MS/MS [46]. Norberto et al. developed a method to analyze paraquat in meconium using GC/MS [20]. HPLC is the most common technique used to analyze paraquat because of its high sensitivity and compatibility for nonvolatile herbicides [47]. However, some of these methods require special equipment or complicated and expensive sample pretreatment, such as solid phase extraction (SPE). In the present study, we describe a modified analytical method to measure paraquat in urine and meconium using potassium ferricyanide for the oxidation of paraquat to form the dipyridone derivative, followed by analysis with high pressure liquid chromatography using a fluorescence detector [48,49].

#### 2.3.1. Chemical Reagents

Paraquat dichloride (1,1′-dimethyl-4,4′-bipyridinium dichloride), ethyl viologen dibromide (1,1′-diethyl-4,4′-bipyridinium dibromide) were used as internal standard (IS), acetonitrile (HPLC grade) and methanol (HPLC grade) were obtained from Sigma-Aldrich (St. Louis, MO, USA). Potassium hydroxide and potassium ferricyanide were obtained from Ajax Finechem (T.S. Interlab Limited Partnership, Bangkok, Thailand). Chloroform was supplied by BDH Prolabo (T.S. Interlab Limited Partnership). Ultrapure water was obtained by Milli-Q system (Millipore, Bedford, MA, USA).

#### 2.3.2. Urine Sample Preparation

The analytical method for urinary paraquat was modified from Tsuchihashi et al. [48] and Blake et al. [49]. This current study analyzed paraquat in urine samples by using the oxidation procedure adapted from Tsuchihashi et al. [48] by adding 3 mL of oxidant (1% of potassium ferricyanide, K_3_Fe(CN)_6_ in 9M-potassium hydroxide (KOH)) in a tube with 3 mL of urine and leaving it to stand for 1 min. A 50 µL aliquot of ethyl paraquat dibromide (6.2 µg/mL) [37,43,44,50] was added as an internal standard (IS). The internal standard used was for correction of the loss of analyte during sample preparation and analysis. Paraquat dipyridone and ethyl paraquat dipyridone were extracted three times with 3 mL chloroform using vortex mixing followed by centrifugation at 4000 rpm for 5 min. The pooled chloroform layer was transferred to a screw capped test tube and evaporated to dryness using nitrogen at room temperature. The residue was reconstituted in 300 µL of methanol/H_2_O (50:50, *v*/*v*), and a 40 µL of the solution was injected into the HPLC system. The ratio of paraquat dipyridone and ethyl paraquat dipyridone was plotted as a calibration curve using paraquat at standard concentrations of 5, 20, 40, 60, 80 and 100 ng/mL spiked in pooled blank urine samples with a coefficient of determination (r^2^) of 0.999. The blank urine sample was collected from unexposed normal population and no parquet was detected in the pooled blank urine. The detection limit was 0.25 ng/mL following the National Institute for Occupational Safety and Health (NIOSH) Method [51]. The limit of detection (LOD) evaluation was carried out by adding additional samples at the low end of the calibration curve (1, 2, 3, 4, 5 ng/mL). These were then plotted and the standard error of the regression was calculated. The LOD was equal to 3 times standard error of the regression divided by the slope of the regression [51]. The QA/QC for the urine samples was prepared as replicate spikes at 30 and 80 ng/mL paraquat and analyzed for recovery and within- and between-day precision on three days. The QA/QC samples were analyzed at the same time as study urine samples and they were within the control limit. The intra- and inter-day variability was carried out at two concentrations for three days analysis. The recovery for between-day was 95.83% and 96.52% and relative standard deviation (RSD) was 3.13% and 4.04% for 30 and 80 ng/mL urine, respectively.

#### 2.3.3. Meconium Sample Preparation

The meconium samples (0.1 g/sample) were homogenized in 3 mL deionized water in a centrifuge tube. A 50 µL of internal standard (6.2 µg/mL) was then added. The tubes were mixed by vortexing, then centrifuged at 6000 rpm for 50 min. The supernatant was transferred to a screw capped test tube. After that, 3 mL of 1% of potassium ferricyanide (K_3_Fe(CN)_6_) in 9M-potassium hydroxide (KOH) was added and left to stand for 1 min. Paraquat dipyridone and ethyl paraquat dipyridone were extracted three times with 3 mL of chloroform using vortex mixing, followed by centrifugation at 4000 rpm for 5 min. The pooled chloroform layer was transferred to a screw capped test tube and evaporated to dryness using a gentle stream of nitrogen at room temperature. The residue was dissolved in 300 µL of methanol/H_2_O (50:50, *v*/*v*), and a 40 µL aliquot of the solution was injected into the HPLC system. The calibration curve of paraquat was set up at 0.155, 0.31, 0.62, 1.24, 1.86 and 2.48 µg/g in pooled blank meconium samples. The blank meconium sample was collected from meconium of infants from unexposed mothers outside the study and no paraquat was detected in the pooled blank meconium. A linear calibration curve was found for the peak area ratio of paraquat dipyridone/ethyl paraqaut dipyridone with a r^2^ of 0.999. The detection limit of paraquat in meconium was 10.9 ng/g. The LOD was done in the same way as urine samples with the spike concentrations added at the low end of the calibration curve being 0.062, 0.093, 0.124, 0.155, 0.186 µg/g. The spiked meconium QA/QC replicate at 0.31 µg/g were analyzed at the same time as the samples and the calibration curve; they were within the control limit. The recovery of meconium for between-day assay was 97.85% and 96.42% and RSD was 1.10% and 3.06% for 0.31 and 1.24 µg/g meconium, respectively.

#### 2.3.4. High Performance Liquid Chromatography Analysis

We modified the HPLC fluorescence method of Blake et al. [49] that used 70% water and 30% acetonitrile as the mobile phase. We used a high performance chromatography (HPLC) system (Agilent 1100 Series, Agilent Technologies (Thailand) Co., Ltd., Bangkok, Thailand) with fluorescence detection (Ex 325 nm, Em 440 nm). The urine and meconium samples were analyzed on a C18 Fortis column (150 mm × 4.6 mm, 5 µm) at 25 °C with a mixture of water (A) and acetonitrile (B) as the mobile phase. The gradient elution was performed as follows: 10% B (initial), 11–17% B (from 2 to 9 min) and 20% B (from 9 to 15 min).

### 2.4. Data Analysis

Descriptive statistics for the urinary paraquat concentrations at the 28th week of pregnancy, delivery and 2 months postpartum and paraquat concentration in meconium were calculated using SPSS (Version 24; PASW Statistic Base 24, SPSS (Thailand) Co., Ltd., Bangkok, Thailand). The chi-square test was used to examine differences in the frequency of agriculturists’ activities between the 28th week of pregnancy and 2 month postpartum. Due to the lognormal distribution of paraquat concentrations in urine and meconium, the paraquat concentrations were reported as the geometric mean, range and geometric standard deviation (GSD). For concentrations below the detection limit, we substituted the detection limit divided by $\sqrt{2}$ [52]. To examine the factors predicting paraquat levels, parametric analyses on the log values were used including t-tests and one-way ANOVAs.

## 3. Results

### 3.1. Demographic Characteristics

The characteristics of the 79 women at the 28th week of pregnancy, delivery and 2 months postpartum are shown in Table 1. The average age was 26 years old (19 to 34). Only 24% graduated from primary school but 64% attended secondary or senior high school. The majority (71%) had farm family members living in the same house and 41% of the women were employed as agriculturists. During pregnancy, approximately half (54%) of the women reported living near farmland (<1 km) where pesticides were sprayed.

### 3.2. Agricultural Activities

During pregnancy, 40% of the pregnant women went to the agricultural fields during the first and/or second trimester of their pregnancy, significantly more than 2 months postpartum. More women reported performing agricultural work during pregnancy than at 2 months postpartum (agricultural work included growing plants, digging in farm soil and picking crops, plants or flowers by hand). More women reported applying herbicides during pregnancy (8%) than at 2 months postpartum (2%). However, reports of paraquat use by the subject or family members were similar for the women during pregnancy (36%) vs. 2 months post-partum (31%) (Table 2).

### 3.3. Paraquat Concentrations in Urine and Meconium Samples

Overall, geometric mean (min-max) of urinary paraquat concentrations among the 79 subjects at the 28th week of pregnancy, delivery and 2 months postpartum were 2.04 (0.18–46.14), 2.06 (0.18–31.96) and 2.42 (0.18–59.86) ng/mL, respectively. However, urinary paraquat concentrations were not significantly different among the three-time sampling points, (*p* = 0.788). The geometric mean (GSD) of urinary paraquat for agriculturists were 1.86 (4.54), 2.01 (5.45) and 2.76 (5.40) ng/mL at the 28th week of pregnancy, delivery and 2 months postpartum, respectively. The geometric mean (GSD) of urinary paraquat among non-agriculturists was 2.18 (4.05), 2.10 (4.87) and 2.16 (5.36) at the 28th week of pregnancy, delivery and 2 months postpartum, respectively (Table 3). 

The urine samples collected at 7th month pregnancy, delivery and 2-months post-partum were not significantly different among the three provinces. The paraquat concentrations in meconium, GM (GSD) were highest at Nakorn Sawan Provinces, 132.46 (5.75) ng/g, followed by Kanchanaburi Province, 33.64 (2.89) ng/g and Amnatchareon Province, 13.12 (3.08) ng/g. Comparisons of the urinary paraquat concentration between those women who were agriculturists compared to those who were not, were not significantly different at any of the sampling time points (Table 3). In all, 28 (55%) of 51 meconium samples were positive for paraquat. Geometric mean (min–max) of the paraquat concentrations in 51 meconium samples was 33.31 (7.70–635.50) ng/g. 

### 3.4. Paraquat Exposure from Agricultural Work Activities

Pregnant women who worked outside their homes had significantly higher urinary paraquat levels at delivery than those worked at home (Table 4). Pregnant women or their family members who used herbicides more than once per crop cycle had urinary paraquat concentrations at delivery significantly higher than those who never used herbicides or used herbicides only once per crop cycle (Table 4). Neonates whose mothers or family members never used herbicides had significantly lower paraquat concentrations in meconium than those who used herbicides. Neonates whose mothers or family members applied paraquat had significantly higher paraquat concentrations in meconium than those who did not. Neonates born to mothers living with family members who were agriculturists had significantly higher paraquat concentrations in meconium than those who did not. Neonates whose mothers lived next to (<1 km) farmland sprayed with pesticides had significantly higher paraquat concentrations in meconium than those who did not. Neonates born to mothers who drank water from community wells had significantly higher paraquat concentrations in meconium than those who drank water from other sources.

## 4. Discussion

Almost one half of the pregnant women in the current study were agriculturists and 11% reported applying pesticides in their fields during pregnancy. These findings were similar to a population of pregnant women in northern Thailand [53]. Usually, Thai women participate in the family business whether it is a grocery store, retail trade or agriculture and they continue working during pregnancy. In this study, the urinary paraquat levels found were very low compared to the levels in pregnant women who used paraquat in a suicide attempt [15,16,17,34,35,36]. Higher concentration of paraquat was seen in post-partum samples compared to 7 months pregnancy and delivery; it could be because the rate of glomerular filtration is high during pregnancy and the rate reduces after delivery for two months [54]. This study does not correct specific gravity or creatinine adjustment; although specific gravity or creatinine adjustment is widely accepted in occupational studies of non-pregnant adults, creatinine adjustment may not be appropriate for metabolite levels in populations undergoing rapid physiologic changes, such as pregnant women and young children, due to high intra-individual variability in creatinine excretion [55]. When urinary paraquat concentrations were compared between agriculturists and non-agriculturists, no significant difference was found. This lack of difference may be for several reasons; only a small number of women reported applying herbicides (2% postpartum and 8% during pregnancy), some women who were not agriculturists lived close to agricultural areas, or lived with family members who were agriculturists and may have been exposed incidentally. In the current study, paraquat in urine could vary by month or crop season depending on types of plants they grow, the length of the growing cycle for each plant type and how often they use paraquat to kill weeds. However, given the study size and the fact that we could only collect a urine sample at 7 months pregnancy, birth and 2 months post-partum; we were limited in our ability to look at the determinants of paraquat exposure. These exposures may also reflect consumption of paraquat residues on fruits and vegetables from the market. The Thai Pesticide Alert Network (Thai-PAN) tested 76 samples of fruits and vegetables in Chiang Mai, Pratumthani, Khon Kaen, Ratchaburi and Songkhla Provinces during August 2017 and found that 55% of fruits and vegetables were contaminated with herbicide residues above maximum residue limit (MRL). 

Moreover, 38 (50%) of the samples had paraquat residues higher than MRL [56]. Furthermore, paraquat residue in dry soybeans was found to be above the MRL when applicators used paraquat at 200 g/rai [57]. Paraquat residues were also found in some Nigerian crops, vegetable and fruits but paraquat residues detected were below MRL [58]. We did find that women who worked outside their home had significantly higher urinary paraquat levels than those who did not. We speculate that this is because women working outside the home were likely to be exposed to paraquat through agricultural activities and contaminated environments at farms or workplaces near agricultural fields. When we classified the frequency of herbicide use by the subject or their family members, we found significant differences in paraquat concentrations at delivery. The higher the frequency of herbicide use, the higher the level of urinary paraquat. Analysis of meconium samples from a clinical study of maternal and fetal exposure to pesticides in the Philippines found that only 2.8% of meconium samples had detectable paraquat concentrations of 0.106 µg/g and 0.046 µg/g [20]. Nevertheless, we detected paraquat in 55% of our samples with a geometric mean (range) of 0.033 (0.007–0.635) µg/g. The limit of detection (LOD) in the Philippines study was 0.0156 µg/g compared with our LOD of 0.0109 µg/g. The higher concentrations measured in this study may be partially attributable to more of the subjects reporting their occupation as agriculturists. In all, 41% of the mothers in this study self-reported their occupation as agriculturists, 71% had farm family living in the same home, 40% worked in an agricultural area during pregnancy and 36% reported that they or their family members used paraquat during their pregnancy.

Unlike the findings for the maternal urinary spot samples taken at three different time periods, the meconium was accumulated starting the third month of pregnancy to delivery period; it is accumulative exposure so would be more difficult to link with paraquat exposure in mothers. A number of factors were significant predictors of neonatal meconium concentrations of paraquat. Neonates born from mothers who applied or whose family members applied herbicides or paraquat during pregnancy had significantly higher paraquat concentrations in meconium than those who did not. Paraquat can cross the placenta to the fetus and accumulate across the gestational period [16,17]. People who lived closer to farmland where pesticides were sprayed had higher urinary metabolites of organophosphate (OP) pesticides than subjects who lived farther away [59,60]. Although urinary levels of paraquat were not significantly different, neonates born to mothers living near pesticide sprayed farmland had higher paraquat concentrations in meconium than those who did not. This may have resulted from the accumulation of exposures over the pregnancy as pesticide vapor and/or droplets present in the air after application were transferred to residential homes close to agricultural areas. Newborns of mothers living with family members who were agriculturists had higher paraquat concentrations in meconium than those who did not. One related study showed that farm children generally had higher estimated pesticide doses than non-farm children, in part because parents who were farmers could transfer OPs to children living in the same house through take-home exposures on their skin and clothing [61].

Neonates born to mothers drinking community well water had significantly higher paraquat concentrations in meconium than those who drank water from other sources. After applying paraquat in the field, the paraquat is degraded in the soil and transported to surface and ground water by agricultural runoff and rain water. Paraquat is highly soluble in water at 20 °C with a pH between 7.2 and 9.2. The solubility in water of paraquat was 620 g/L [62] and the half-life of paraquat in water was between 2 to 820 years [3]. Paraquat concentrations have been found in groundwater >18.9 mg/L in the Yom River Basin in Thailand [25]. Paraqaut concentrations in the Chanthaburi River in dry and wet seasons ranged from 0.13 to 7.13 and 0.07 to 13.05 µg/L, respectively [23]. These studies showed that both ground and surface water were contaminated with paraquat. In these areas people who drink or use contaminated ground and surface water can be exposed to paraquat.

Exposure to paraquat during pregnancy may increase the risk of adverse health effects in mothers and their neonates. The target organs of paraquat include the eyes, skin, respiratory system, heart, liver, kidneys and gastrointestinal tract [63]. Exposure to paraquat over a long period increases the risk of Parkinson disease [9,10]. Paraquat, a dopaminergic neurotoxin, can cross the blood-brain barrier [64] and accumulate in the frontal cortex, striatum, hippocampus and cerebellum [65,66]. Exposure to 20 mg/kg of paraquat during pregnancy in mice showed decreased levels of locomotor activity and cognitive function in neonates [67]. Chronic prenatal exposure to paraquat could alter a developing mouse brain and its functions, causing impairment of motor coordination that may become apparent with advancing age [18]. However, no research has investigated the developmental effects of paraquat on humans; therefore, chronic health effects of exposure to paraquat while in-utero need more investigation.

The strengths of this study are: the detection limit of the methods used to measure paraquat in urine and meconium were very low. It is the first study looking at paraquat that collected urine samples in three time periods 7 months’ pregnancy, delivery and 2 months postpartum and meconium samples from the newborn infants among women in Thailand who are both agriculturists and non-agriculturists, enabling us to compare the biomarker levels in these groups. The weaknesses of this study are: the number of subjects is small and with urine samples collected at only 3 time periods per subject, limiting the analysis of exposure determinants. The urine and meconium samples were kept at −45 °C for 5 years before analysis, and we do not know if some sample degradation occurred during this time. Lastly, questionnaires were used to collect exposure determinant information from the pregnant women, but we also asked them to answer questions related to family members who worked in agriculture. This second hand information may have been less reliable.

## 5. Conclusions

The urinary paraquat concentrations of pregnant women living in an agricultural area was similar at 28 weeks of pregnancy, delivery and 2 months postpartum. However, certain agricultural activities resulted in significantly higher urinary concentrations of paraquat. In our study, 55% of newborn meconium samples had measureable paraquat concentrations. Pregnant women living near farmland sprayed with pesticides, having family members who are agriculturists living in the same home, drinking well water, using herbicides and using paraquat resulted in significantly increased the levels of paraquat in the meconium of neonates. Because meconium accumulates over the pregnancy, it is not known when exposure occurred. Exposures during different time windows are known to differentially impact fetal development. Due to the risk of maternal and fetal exposures to this toxic pesticide and links with chronic diseases such as Parkinson’s and cancer, Thailand should consider ways to limit the availability of paraquat for use by Thai agriculturists.

## Figures and Tables

**Table 1:** Demographic characteristics of pregnant women at 28 weeks of pregnancy (*n* = 79).

Characteristics of Pregnant Women	28 Weeks of Pregnancy
	Number (%)
**Age (years)**	
Mean (SD) years	25.5 (4.3)
Range (years)	19–34
**Education level**	
Primary school or lower	19 (24)
Junior high school	31 (39)
Senior high school or vocational certificate	20 (25)
College certificate or high vocational certificate	7 (9)
Bachelor degree	2 (3)
**Occupation**	
Agriculturists	32 (41)
Other	47 (59)
**You have farm family**	
Yes	56 (71)
No	23 (29)
**Live next to farmland where pesticides are sprayed**	
Not near farmland	36 (46)
Near farmland where pesticides are sprayed	42 (54)

**Table 2:** Agricultural activities of women at 28 weeks of pregnancy and 2 months postpartum.

Agricultural Activities of Women	28th Week of Pregnancy *n* (%)	2 Months Postpartum *n* (%)	*p*-Value
**Visit agricultural fields**			
Yes	30 (40)	3 (5)	<0.001 *
No	46 (60)	56 (95)	
**Farm work related to growing plants**			
Yes	18 (25)	1 (2)	<0.001 *
No	55 (75)	61 (98)	
**Apply chemical fertilizer, manure or compost**			
Yes	14 (19)	3 (5)	<0.001 *
No	59 (81)	59 (95)	
**Dig in farm soil**			
Yes	14 (19)	1 (2)	<0.001 *
No	59 (81)	61 (98)	
**Apply pesticides**			
Yes	8 (11)	0 (0)	0.008 *
No	65 (89)	62 (100)	
**Apply herbicide to control weeds**			
Yes	6 (8)	1 (2)	0.031 *
No	67 (92)	61 (98)	
**Hand**-**pick crops, plants or flowers**			
Yes	19 (26)	2 (3)	<0.001 *
No	54 (74)	60 (97)	
**You or family members use paraquat**			
Yes	24 (36)	17 (31)	0.146
No	43 (64)	38 (69)	

* Significant at criterion of *p* < 0.05.

**Table 3:** Urinary paraquat concentrations at three time points: 28th weeks of pregnancy, delivery and 2 months postpartum and paraquat concentrations in meconium of neonates.

Parameter	Agriculturists	Non-Agriculturists	*p*-Value
**Urine at 28th Week of Pregnancy**	*n* = 32	*n* = 47	
Detection frequency (%)	26 (81)	39 (83)	
GM (GSD) ng/mL	1.86 (4.54)	2.18 (4.05)	0.632
**Urine at Delivery**	*n* = 30	*n* = 47	
Detection frequency (%)	22 (73)	38 (81)	
GM (GSD) ng/mL	2.01 (5.45)	2.10 (4.87)	0.915
**Meconium of neonates**	*n* = 22	*n* = 29	
Detection frequency (%)	11 (50)	17 (59)	
GM (GSD) ng/g	29.56 (4.69)	36.48 (4.62)	0.630
**Urine at 2 months postpartum**	*n* = 28	*n* = 34	
Detection frequency (%)	23 (82)	25 (73)	
GM (GSD) ng/mL	2.76 (5.40)	2.16 (5.36)	0.570

**Table 4:** Urinary paraquat concentration of women at 28th week of pregnancy, and delivery and paraquat concentration in meconium samples categorized by exposure factors.

Activities Reported by Women	Prenatal *n* = 79 (%)	Prenatal GM (GSD)	Birth *n* = 77 (%)	Birth GM (GSD)	Meconium *n* = 51 (%)	Meconium GM (GSD)	2 Month Postpartum *n* = 62 (%)	2 Month Postpartum GM (GSD)
**Have household family members who are agriculturists**								
Yes	56 (71)	2.11 (4.65)	54 (71)	1.96 (5.8)	38 (75)	42.09 (5.10)	46 (74)	2.13 (5.45)
No	23 (29)	1.87 (3.29)	23 (29)	2.33 (3.49)	13 (25)	16.81 (2.47)	16 (26)	3.45 (4.97)
*p*-value		0.729		0.629		0.016 *		0.326
**Worked outside your home**								
Yes	39 (51)	1.78 (4.27)	37 (49)	3.2 (5.1)	20 (40)	32.49 (5.26)	15 (25)	3.33 (5.1)
No	38 (49)	2.22 (4.27)	38 (51)	1.38 (4.39)	30 (60)	33.88 (4.42)	46 (75)	2.16 (5.52)
*p*-value		0.504		0.021 *		0.926		0.392
**Drink water from community well**								
Yes	9 (12)	1.63 (4.23)	9 (12)	1.82 (5.02)	7 (14)	124.9 (3.35)	8 (13)	1.11 (5.09)
No	69 (88)	2.06 (4.25)	67 (88)	2.17 (5.01)	44 (86)	26.99 (4.37)	53 (87)	2.75 (5.35)
*p*-value		0.651		0.756		0.012 *		0.121
**Live next to farmland where pesticides are sprayed**								
Yes	42 (54)	1.72 (4.11)	41 (54)	1.86 (5.71)	29 (43)	49.62 (4.79)	34 (56)	2.26 (5.09)
No	36 (46)	2.39 (4.34)	35 (46)	2.50 (4.17)	22 (57)	19.70 (3.77)	27 (44)	2.68 (5.94)
*p*-value		0.313		0.43		0.031 *		0.697
**You or your family applied herbicides**								
Yes	43 (58)	2.39 (4.69)	41 (57)	2.31 (5.8)	29 (62)	50.99 (4.05)	34 (59)	2.24 (5.25)
No	31 (42)	2.03 (3.19)	31 (43)	1.86 (3.91)	18 (38)	13.53 (2.32)	24 (41)	3.63 (5.13)
*p*-value		0.601		0.578		0.001 *		0.36
**You or family members use paraquat**								
Yes	24 (36)	2.53 (4.43)	22 (34)	2.24 (6)	17 (40)	73.29 (4.72)	17 (31)	1.48 (5.40)
No	43 (64)	1.96 (3.59)	43 (66)	2.27 (4.58)	26 (60)	19.07 (3.39)	38 (69)	2.56 (5.34)
*p*-value		0.467		0.976		0.003 *		0.268
**How often do you or your family members use herbicides per crop cycle?**								
A1. No	31 (42)	2.13 (3.23)	31 (43)	1.86 (3.91)	18 (38)	13.53 (2.32)	24 (41)	3.36 (5.13)
A2 Once per crop cycle	24 (32)	1.87 (3.91)	24 (33)	1.31 (4.91)	19 (41)	44.79 (4.28)	18 (31)	2.83 (4.78)
A3. More than once per crop cycle	19 (26)	2.52 (4.54)	17 (24)	5.13 (5.53)	10 (21)	65.21 (6.71)	16 (28)	1.72 (5.87)
*p*-value for one way ANOVA		0.908		0.02 *		0.008 *		0.451
and all pairwise comparison				A1 vs. A3 *p* = 0.032 * A2 vs. A3 *p* = 0.006 * A1 vs. A2 *p* = 0.399		A1 vs. A2 *p* = 0.011 * A1 vs. A3 *p* = 0.006 * A2 vs. A3 *p* = 0.487		

* Significant at criterion of *p* < 0.05.
